# Selfish bacteria are active throughout the water column of the ocean

**DOI:** 10.1038/s43705-023-00219-7

**Published:** 2023-02-04

**Authors:** Greta Giljan, Sarah Brown, C. Chad Lloyd, Sherif Ghobrial, Rudolf Amann, Carol Arnosti

**Affiliations:** 1 Department of Molecular Ecology, Max Planck Institute for Marine Microbiology, Bremen, Germany; 2 Environment, Ecology, and Energy Program, University of North Carolina-Chapel Hill, Chapel Hill, NC USA; 3 Department of Earth, Marine, and Environmental Sciences, University of North Carolina-Chapel Hill, Chapel Hill, NC USA

**Keywords:** Water microbiology, Biogeochemistry

## Abstract

Heterotrophic bacteria in the ocean invest carbon, nitrogen, and energy in extracellular enzymes to hydrolyze large substrates to smaller sizes suitable for uptake. Since hydrolysis products produced outside of a cell may be lost to diffusion, the return on this investment is uncertain. Selfish bacteria change the odds in their favor by binding, partially hydrolyzing, and transporting polysaccharides into the periplasmic space without loss of hydrolysis products. We expected selfish bacteria to be most common in the upper ocean, where phytoplankton produce abundant fresh organic matter, including complex polysaccharides. We, therefore, sampled water in the western North Atlantic Ocean at four depths from three stations differing in physiochemical conditions; these stations and depths also differed considerably in microbial community composition. To our surprise, we found that selfish bacteria are common throughout the water column of the ocean, including at depths greater than 5500 m. Selfish uptake as a strategy thus appears to be geographically—and phylogenetically—widespread. Since processing and uptake of polysaccharides require enzymes that are highly sensitive to substrate structure, the activities of these bacteria might not be reflected by measurements relying on uptake only of low molecular weight substrates. Moreover, even at the bottom of the ocean, the supply of structurally-intact polysaccharides, and therefore the return on enzymatic investment, must be sufficient to maintain these organisms.

## Introduction

High molecular weight carbohydrates—polysaccharides—constitute a major fraction of phytoplankton and of detrital organic matter in the ocean [1, 2]. The degradation of polysaccharides depends largely on the activities of bacteria equipped with the extracellular enzymes required to dismantle these often highly complex structures to low molecular weight pieces (e.g., [3]). Production of extracellular enzymes requires a substantial investment of carbon, nitrogen, and energy on the part of bacteria, particularly since a large number of structurally selective enzymes may be required to hydrolyze a single polysaccharide [3, 4]. Since low molecular weight hydrolysis products may also be taken up by “scavengers” that do not produce the enzymes, the enzyme “producers” that carry out external hydrolysis might benefit only in part from their own enzyme activities [5]. However, an additional strategy of high molecular weight organic matter utilization, “selfish” uptake [6], is used for polysaccharide degradation, and has recently been found to be widespread among bacterial communities in surface ocean waters [7–9]. During selfish uptake, polysaccharides are bound at the cell surface, partially hydrolyzed, and transported into the periplasmic space without substantial loss of hydrolysis products [6], thereby retaining hydrolysate for the selfish bacteria and reducing the availability of low molecular weight substrates to scavenging bacteria [10]. Instead of a two-player model consisting of producers and scavengers [5], then, polysaccharide degradation by bacteria should be considered in the framework of a three-player model: the selfish bacteria and producers that both focus on degradation of polysaccharides, as well as the scavenging bacteria that may benefit from external hydrolysis carried out by the producers [10].

Quantifying the relative contributions of these mechanisms of polysaccharide processing to carbon cycling in the ocean requires an understanding of their spatial and temporal extent, as well as of the factors that may favor one mechanism of polysaccharide processing over the other. To date, investigations have demonstrated that a broad range of polysaccharides is taken up via selfish mechanisms by diverse bacteria in surface waters of the Atlantic and Pacific Oceans [7–9, 11, 12]. The speed and extent of selfish uptake and external hydrolysis vary by geographic location as well as by specific polysaccharide [8, 9]; initial population size and relative growth rate of selfish bacteria and external hydrolyzers likely affect the balance of these mechanisms [8]. The observation that the extent of selfish uptake and external hydrolysis changes during different phytoplankton bloom stages—and varies with polysaccharide structural complexity [11, 12]—suggests that dynamic interaction of a wide range of organisms and highly diverse substrate structures governs polysaccharide processing in sunlit surface ocean waters.

The extent to which selfish bacteria may be present and active in other depths of the ocean, however, remains unexplored. Given that polysaccharide-hydrolyzing enzymes are exquisitely specific for substrate structure [13], we hypothesized that selfish bacteria, which require multiple interacting enzymes, would be most abundant and active in locations and at depths at which freshly produced—structurally unaltered—polysaccharides are common. In particular, we expected that selfish bacteria would be particularly dominant in the upper water column since high molecular weight dissolved organic matter as well as particulate organic matter are far more abundant and are “fresher”—have a higher fraction of chemically characterizable components—in the upper ocean than in the deep ocean [14, 15].

To test this hypothesis, we sampled water at three stations characterized by different physical, chemical, and productivity conditions in the western North Atlantic: in the Gulf Stream, in productive waters off of the coast of Newfoundland, and in the oligotrophic waters of the North Atlantic Gyre (Fig. S1). At these stations, we collected water from the surface, deep chlorophyll maximum (DCM; 33–104 m), upper mesopelagic (~300 m), and bottom (3190–5580 m). Triplicate incubations were made with water from three different Niskin bottles from each depth. We quantified the presence and activity of selfish bacteria by adding small quantities of structurally distinct fluorescently-labeled polysaccharides (FLA-PS) and incubating these water samples at in situ temperatures. The added FLA-PS—laminarin, pullulan, fucoidan, xylan, chondroitin sulfate, and arabinogalactan—have different monomer compositions and linkage types. These polysaccharides were chosen because they are abundant in marine algae and phytoplankton and/or because a wide range of marine bacteria may produce enzymes that hydrolyze them (e.g., [16–20]). We concurrently measured selfish uptake and external hydrolysis rates of the FLA-PS, quantified cell abundances, measured bacterial protein production, and tracked bacterial community composition to investigate the activities of heterotrophic bacteria and their different modes of polysaccharide processing throughout the water column.

## Materials and methods

### Station location and seawater collection

Seawater was collected at three stations in the western North Atlantic aboard the research vessel *Endeavor* (cruise EN638) using a sampling rosette of 30-l Niskin bottles fitted with a Sea-Bird 32 conductivity-temperature-depth (CTD) profiler, between May 15th and 30th 2019 (Fig. S1). Collection depths included surface water (2.5–6 m water depth), the deep chlorophyll maximum (DCM; depth identified via chorophyll fluorescence signal of the CTD: 104 m, 33 m, 64 m water depth at Stns. 18, 19, and 20, respectively), ~300 m (300 m at Stns. 18 and 20; 318 m at Stn. 19), and bottom water (3 190 m, 4 325 m, and 5 580 m, at Stns. 18, 19, and 20, respectively; Fig. S1).

At each station and depth, triplicates of 600 mL (DCM and bottom water) or 290 mL (surface and 300 m water) were added to sterile, acid rinsed (10% HCl) bottles and incubated for up to 30 days in the dark at in situ temperatures (room temperature for water from the surface, DCM and 300 m; 4 °C for bottom water) with one of the six FLA-PS: arabinogalactan, chondroitin sulfate, laminarin, pullulan, and xylan, each at 3.5 µM monomer equivalent concentration, and fucoidan at 5.0 µM monomer equivalent concentration, due to its low labeling density. A single live treatment control without the addition of any substrate was included for the DCM and bottom waters; autoclaved killed controls were included for each substrate at each station and each depth, and were incubated under the same conditions alongside polysaccharide incubations.

Subsamples for microbial cell counts and selfish FLA-PS uptake were collected from DCM and bottom water incubations 0, 1, 3, 7, and 10 days after the addition of polysaccharides; in surface and 300 m incubations, subsamples were collected 0, 3, 7, 10, and 15 days after polysaccharide addition. Note also that the t0 timepoint measurements of selfish uptake represent a time period of ca. 30 min (surface, 300 m) to 5 h (DCM, bottom water), due to the processing time required after initial substrate addition. To measure the extracellular hydrolysis of FLA-PS, subsamples were collected on days 0, 3, 7, 10, 15, and 30 of the incubations. Subsamples for bulk community analysis were taken before the addition of FLA-PS and at day 1, 3, and 10 of the incubation with DCM and bottom water and at day 0, 3, 7, 10, and 15 in the surface and 300 m incubations.

### Synthesis of FLA-PS and measurements of extracellular enzymatic activities

Arabinogalactan, chondroitin sulfate, fucoidan, laminarin, pullulan, and xylan were fluorescently labeled with fluoresceinamine (Sigma) and characterized according to Arnosti [21]. Subsamples (2 ml) from the filtrate of the samples collected for taxonomic community analysis (see below) were collected at days 0, 3, 7, 10, 15 post FLA-PS addition; an additional sample was collected from the incubation flasks and filtered through a 0.2 μm pore-sized filter at 30 days. These samples were analyzed after Arnosti (2003) [21] to determine the extracellular (external) hydrolysis of high molecular weight polysaccharides to low molecular weight hydrolysis products. Note that the added substrate is in competition with naturally occurring substrates, and thus calculated hydrolysis rates represent potential hydrolysis rates.

### Counts of total and substrate-stained cells

To prepare samples for microscopic quantification, 25–50 mL of the substrate incubations were fixed with a final concentration of 1% formaldehyde overnight and subsequently filtered onto 0.22 µm pore size polycarbonate filters at a maximum vacuum of 200 mbar. The DNA of filtered cells was counterstained using 4′,6-diamidin-2-phenylindol (DAPI) and mounted with a Citifluor/VectaShield (4:1) solution. A minimum of 45 microscopic images per sample were acquired as described by Bennke et al. [22] with a fully automated epifluorescence microscope (Zeiss AxioImager.Z2 microscope stand, Carl Zeiss) equipped with a cooled charged-coupled-device camera (AxioCam MRm + Colibri LED light source, Carl Zeiss), three light-emitting diodes (UV-emitting LED, 365 nm for DAPI; blue-emitting LED, 470 nm for FLA-PS 488) and a HE-62 multi filter module with a triple emission filter (425/50 nm, 527/54 nm, LP 615 nm, including a triple beam splitter of 395/495/610, Carl Zeiss) using a 63× magnification oil immersion plan apochromatic objective with a numerical aperture of 1.4 (Carl Zeiss). Final cell enumeration on the acquired images was performed using the image analysis software ACMETOOL (http://www.technobiology.ch and Max Planck Institute for Marine Microbiology, Bremen). Automated cell counts were checked manually.

The numbers of total microbial cells and FLA-PS stained cells were counted in a single experimental setup, following Reintjes et al. [7]. Selfish substrate uptake could be measured for only four or five of the six polysaccharides used. At all stations and depths, xylan incubations yielded high background fluorescence, which interfered with cell counting; this problem also affected efforts to count cells for pullulan uptake in surface waters and at a depth of 300 m. Note also that we report the fraction of cells carrying out selfish uptake under the assumption that each substrate is taken up by different bacteria (i.e., when reporting that for example, 22% of total DAPI-stainable cells were substrate-stained, we add together the percentages taking up laminarin, fucoidan, arabinogalactan, and chondroitin). Since selfish uptake of each substrate is measured in different incubations (triplicate incubations of each individual substrate), however, it is possible that some or all of the cells taking up one substrate also take up another substrate via a selfish mechanism.

### Super-resolution imaging of selfish polysaccharide uptake

The specific substrate accumulation pattern in FLA-PS stained cells was visualized on a Zeiss LSM780 with Airyscan (Carl Zeiss) using a 405 nm, a 488 nm, and a 561 nm laser with detection windows of 420–480 nm, 500–550 nm, and LP 605 nm, respectively. Z-stack images of the cells were taken with a Plan-Apochromat 63×/1.4 oil objective and the ZEN software package (Carl Zeiss) was used for subsequent AiryScan analysis.

### Taxonomic bacterial community analysis

The initial bacterial community composition in each sample and changes over the course of the incubation were determined through 16S rRNA analysis. For this analysis, 25 mL samples from each incubation were filtered onto a 0.22 µm pore size polycarbonate filter at a maximum vacuum of 200 mbar, dried and frozen at −20 °C until further processing. Total DNA extraction from filter was done using the DNeasy Power Water Kit (Quiagen). Determination of the concentration as well as the size of the extracted DNA was done via gel chromatography using a Fragment Analyzer^TM^ (Advanced Analytical). Amplification of the variable 16S rRNA regions V3 and V4 (490 bp) was done in 30 cycles using the 5 PRIME HotMasterMix (Quantabio) together with the Bakt_314F (CCTACGGGNGGCWGCAG) and Bakt_805R (GACTACGVGGGTATCTAATCC) [23] PCR primer pair with an individual 8 bp barcode adapter (based on the NEB Multiplex Oligos for Illumina, New England Biolabs) attached to the forward primer and the reverse primer. The amplified PCR product was purified and size selected using the AMPure XP PCR Cleanup system (Beckman Coulter). Barcoded products were pooled in equimolar concentrations and sent for paired-end Illumina sequencing (2 × 250 bp HiSeq2500) to the Max Planck-Genome-center Cologne. Sequences were merged, demultiplexed and quality trimmed (sequence length 300–500 bp, <2% homopolymers, <2% ambiguities) with BBTools [24]. The SILVAngs pipeline [25] with the SSU rRNA SILVA database 138 was used for sequence comparison and taxonomic assignment of the retrieved sequences.

### Statistical analysis of bacterial communities

Analysis of the bacterial community composition was done using normalized reads representing >1000 reads per sample. The average of triplicates for each FLA-PS amended incubation was used for further analysis and visualization. Archaeal and eukaryal reads were excluded from analysis. Differences in community composition between stations, water depths, incubation times, and substrates among amended and unamended incubations were analyzed by analysis of similarity and visualized in nonmetric multi-dimensional scaling plots, using Bray–Curtis dissimilarity matrices. Community shift over the course of the incubations was visualized by comparison of the read abundance on genus level from the initial community with the read abundance in the incubations at each time point.

### Bacterial productivity

Bacterial productivity was measured after Kirchman et al. [26]. In brief, bacterial protein production was calculated from leucine incorporation rates, measured in samples that were incubated at in-situ temperatures in the dark for time periods of 12–24 h. Bacterial carbon production was calculated by multiplying bacterial protein production by 0.86 [26, 27].

## Results

**Fig. 1:**
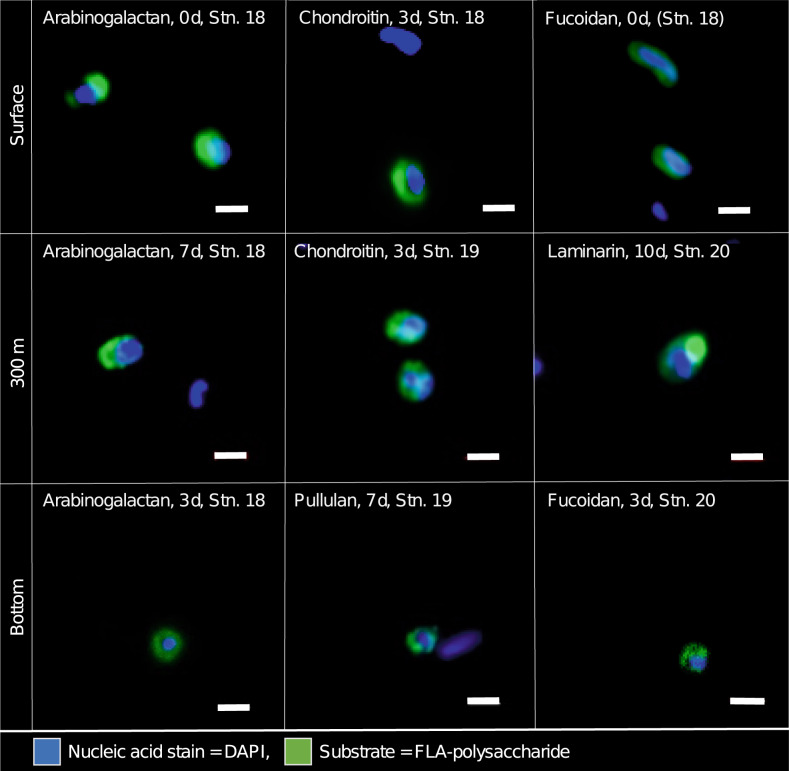
Selfish bacteria throughout the water column in the western North Atlantic Ocean. Super-resolution images of microbial cells from surface water, 300 m water depth, and bottom water (3 190, 4 325, and 5580 m, at Stns. 18, 19, and 20, respectively) showing accumulation of fluorescently labeled arabinogalactan, fucoidan, chrondroitin sulfate, laminarin, and pullulan due to selfish uptake. Blue signal (DAPI) shows nucleic acids, green signal is due to fluoresceinamine-labeled polysaccharides. Scale bar = 1 μm.

**Fig. 2:**
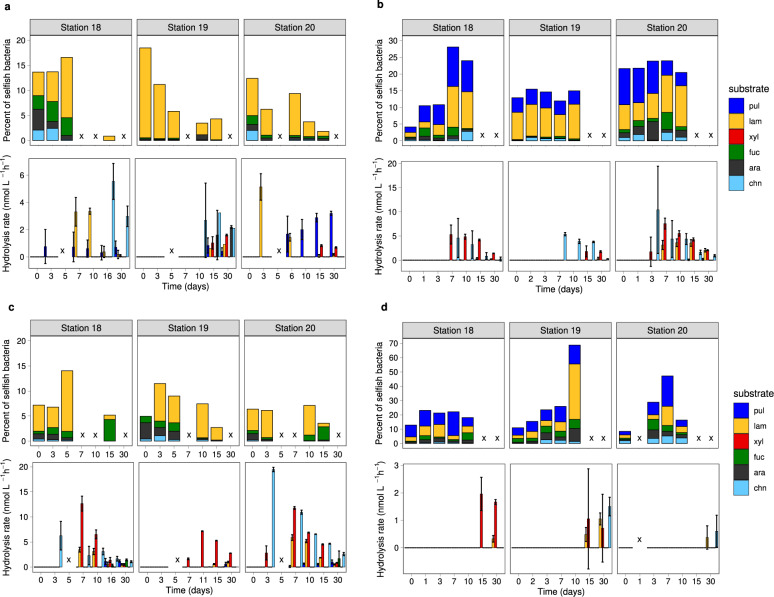
Heterotrophic polysaccharide utilization throughout the water column at three distinct locations in the western North Atlantic Ocean. Selfish uptake and extracellular (external) hydrolysis of six different fluorescently-labeled polysaccharides (FLA-PS) in **a** surface waters, **b** at the deep chlorophyll maximum (DCM), **c** at 300 m water depth, and **d** in bottom waters at Stns. 18, 19, and 20 over the course of individual FLA-PS amended incubations. Note that the scales of the *Y*-axes vary by depth. Note also that selfish FLA-xylan uptake at all stations and FLA-pullulan uptake in surface waters and at 300 m depth could not be analyzed due to high background fluorescence, and are therefore not included in the data. Error bars represent the average of biological replicates (*n* = 3). Samples marked with an x were not analyzed.

Much to our surprise, selfish bacteria were abundant at all water depths that we investigated. These bacteria were identified microscopically by the co-localization of the blue DAPI staining of DNA and the associated intense green staining from the FLA-PS (Fig. 1). Selfish uptake of laminarin was substantial, especially in surface waters of Stn. 19, but structurally much more complex substrates such as fucoidan [4] also contributed notably to selfish uptake (Fig. 2). Overall, a broad range of substrates was taken up at almost every depth and station.

The considerable selfish activity was evident even at the t0 timepoint, when 14–17% of bacteria were in surface water, 5–22% at the DCM, 5–8% at 300 m, and 5–12% of bacteria in bottom water took up one of the FLA-PS (Fig. 2). With increasing incubation time, the proportion of cells taking up one of the FLA-PS increased, especially in sub-surface waters. Selfish uptake reached a maximum of 13–18% of DAPI-stainable cells in surface waters, 14–26% at the DCM, 12–18% at 300 m, and 25–67% in bottom water. Uptake at the t0 timepoint reflects the short-term response of the in situ community, since the time elapsed between substrate addition and sample processing is likely insufficient for major changes in community composition, while later timepoints reflect the activities of a community that has changed in composition with time.

**Fig. 3:**
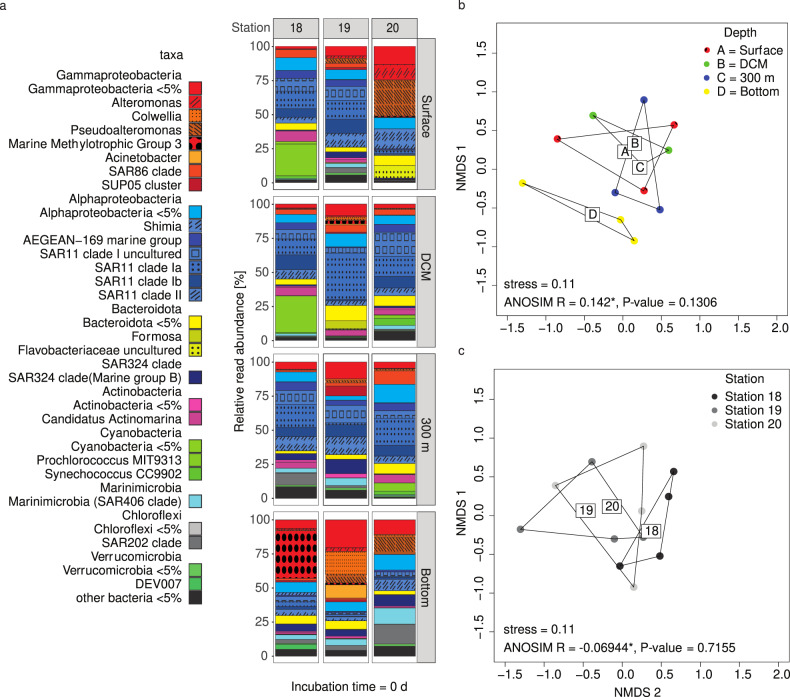
The initial bacterial communities at four depths at three distinct locations in the western North Atlantic Ocean differed considerably from one another. Bacterial communities (**a**) differ more by depth (**b**) but are also different by station (**c**). * denotes statistically significant results. Each community represents the average of biological replicates (*n* = 3).

**Fig. 4:**
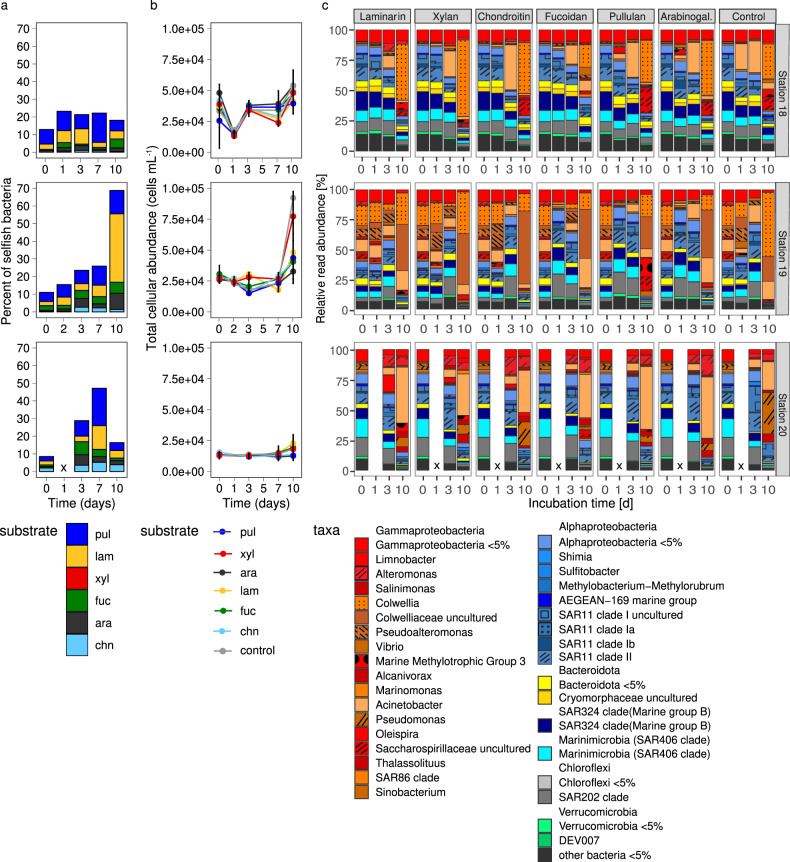
Selfish polysaccharide uptake, cell counts, and bacterial community composition in North Atlantic bottom water. **a** Selfish uptake of FLA-PS over the course of 10-day FLA-PS amended incubations at Stns. 18, 19, and 20 (same data as in Fig. 2d). **b** Development of total microbial cell counts and **c** bacterial community composition in each of the FLA-PS amended incubations from **a**, and in the unamended treatment control. The initial community shortly after the addition of the FLA-PS is depicted at 0 days of incubation. Error bars represent the average of up to three replicates. Time points marked with an x were not analyzed.

Substantial selfish uptake characterized the in situ bacterial community despite initial station- and depth-related differences in composition (Fig. 3). Moreover, although these initial communities changed markedly for the most part over the time course of incubation (Fig. 4; Figs. S2–S5), the percentage of the community carrying out selfish uptake at most stations and depths remained constant or increased after the t0 timepoint. The compositional changes in unamended incubations were very similar to the incubations amended with FLA-PS, demonstrating that the addition of FLA-PS by themselves had little influence on community composition (Figs. Figs. S2c–S5c) or cell counts (Fig. S6). Furthermore, the changes in community composition typically were not convergent for different depths (Fig. S7), in that different genera dominated even in cases where similar classes became more abundant with time. For example, although Gammaproteobacteria became relatively more abundant with time in many of the incubations (Fig. 4, Figs. S2–S5), the dominant phylotypes varied by depth and station (Figs. S8–S10). In sum, there were no obvious connections between specific changes in bacterial community composition and selfish uptake.

We observed remarkable differences in patterns of selfish uptake and external hydrolysis, which were measured in the same incubation containers. While selfish uptake was measurable for a broad range of polysaccharides at all stations and most depths, external hydrolysis was more variable among stations at similar depths, and decreased sharply in bottom water compared to surficial waters (Fig. 2). In surface waters at Stn. 19, for example, all polysaccharides except fucoidan were externally hydrolyzed, but at Stns. 18 and 20 only laminarin, chondroitin, and xylan were externally hydrolyzed; these spatial variations in external hydrolysis are consistent with previous observations in surface ocean waters [28]. Station-related variability was also evident at a depth of 300 m: at Stns. 18 and 20, a broad spectrum of polysaccharides was externally hydrolyzed, but at Stn. 19, only xylan, laminarin, and pullulan were externally hydrolyzed. In bottom waters of all three stations, only two or three polysaccharides were externally hydrolyzed—laminarin and xylan at Stn. 18, laminarin, xylan, and chondroitin at Stn. 19, and laminarin and chondroitin at Stn. 20. Those polysaccharides were hydrolyzed at comparatively low rates, and hydrolysis was first evident at late incubation timepoints. Overall, the spectrum of substrates externally hydrolyzed was narrower and the hydrolysis rates in deep water were considerably lower than in surface water, consistent with the few previous reports of polysaccharide hydrolysis in deep ocean waters [29–31]. Patterns of external hydrolysis and selfish uptake at the same stations and depths thus showed striking contrasts.

## Discussion

The presence of selfish bacteria in the water column, also at depths well below the euphotic zone, requires reconsideration of mechanisms, strategies, and economics of substrate processing, bacterial physiology, and the role of high molecular weight organic matter in fueling bacterial metabolism. Although selfish uptake mechanisms have been studied most intensively in members of the gut-dwelling Bacteroidetes [32, 33], selfish activity is also common in surface marine waters [7–9, 11, 12]. Our previous investigations demonstrated that a range of bacteria, including members of the Bacteroidetes, Planctomycetes, Verrucomicrobia, and the genus *Catenovulum* (Gammaproteobacteria) carry out selfish uptake [8, 12]. Furthermore, a large fraction of selfish bacteria is still unidentified [8, 12]. The observation of selfish activity at multiple depths throughout the water column against a background of changing bacterial community composition suggests that this strategy of substrate acquisition is widespread, and in the ocean is not restricted to a limited range of bacterial taxa. Since selfish uptake cannot be inferred solely from genomic information [34], however, we cannot yet determine whether selfish uptake mechanisms are as widely distributed as the ability to produce extracellular enzymes to carry out external hydrolysis.

The economics of substrate processing and uptake also should be reconsidered. Two-player models of enzyme producers and scavengers have considered the conditions under which extracellular enzyme production may pay off (e.g., [5]), such as when polysaccharides are abundant [35], or when they are found in sufficiently dense patches [36]. Inclusion of selfish bacteria in this calculus, as described in a new conceptual model [37], suggests that substrate structural complexity, as well as abundance, needs to be taken into account. Selfish uptake, in which most hydrolytic activity occurs in the periplasm—between outer and inner membranes—prevents diffusive loss of hydrolysate, and ensures that investment in extracellular enzymes generates sufficient return. From this perspective, selfish uptake appears to be widespread either i) when a substrate is highly complex and requires considerable enzymatic investment, or ii) when there is high competition for a very widely-available substrate, such that competition is a primary consideration [37].

The first case covers enzymatic investment to acquire comparatively rare and structurally complex substrates that are selfishly taken up, since the number of different enzymes required for hydrolysis scales directly with polysaccharide structural complexity [38]. Arguably, intact polysaccharides are likely a comparatively rare commodity in most of the subsurface ocean, including the deep ocean (Fig. 4). Any unaltered polysaccharides thus should be a target for selfish uptake, a consideration that would explain the broad range of substrates selfishly taken up in much of the water column. Polysaccharides such as fucoidan are particularly good examples of a potential pay-off from selfish uptake since fucoidan hydrolysis requires an extraordinary investment in enzymes [4]. Moreover, external hydrolysis of fucoidan is comparatively rarely detected in the surface ocean [28], and to date has not been detected in deep ocean waters [29–31, 39].

The second case for which selfish uptake pays off—high competition for an abundant polysaccharide—applies especially to laminarin. Oceanic production of laminarin has been estimated to be on the order of 12–18 gigatons annually [16, 40], providing a vast supply of readily-degradable substrate to heterotrophic microbial communities. Moreover, external hydrolysis of laminarin is measurable in almost every site and location in the ocean investigated to date [7–9, 11, 12, 28–31, 39], pointing at very widespread capabilities to utilize this polysaccharide. Selfish uptake of laminarin, therefore, ensures return on enzyme investment by capturing a substrate that would otherwise be acquired by competitors.

Rapid selfish uptake of polysaccharides, especially in bottom water, also provides important clues about the physiology of bacteria in the deep ocean, and the nature of the substrates that they use. Approximately 5–10% of the bacterial community at our three deep ocean sites was ready and able to take up specific polysaccharides shortly after addition (Figs. 1, 2 and 4). This response is notable in light of the observation that uptake of a simple amino acid at these depths (as demonstrated by leucine used for bacterial productivity measurements; Table S1), which does not require prior enzymatic hydrolysis, was quite low especially at Stns. 18 and 19. These observations together suggest that a bacterial strategy focused on rapid uptake of structurally more complex, higher molecular weight polysaccharides pays off in deep water because there is a sufficient supply of these substrates, whereas the in situ inventory of individual amino acids in bottom water is likely too low [41] to merit special targeting by bacteria.

Although we currently lack data on the polysaccharide component of particulate organic matter in bottom waters, a new method to specifically quantify laminarin in particles has demonstrated a considerable laminarin concentration in particulate organic matter in the upper water column (including measurements to a depth of 300 m [40]). Furthermore, rapid transport of bacteria/particles—including intact diatom cells [42]—to bottom water depths has been demonstrated [43, 44]. This organic matter likely includes intact polysaccharides such as laminarin and fucoidan [45]. Recent measurements of dissolved organic matter in deep ocean waters additionally suggest that polysaccharides derived from the surface ocean are added to the dissolved organic matter pool circulating in the deep ocean [46].

Selfish uptake of highly complex polysaccharides in the deep ocean thus may be supported by a substantial flux of relatively unaltered organic matter to the deep ocean [47, 48]. The presence of bacteria that are capable of processing complex substrates in a selfish manner also suggests that measurements of bacterial metabolism that are dependent upon uptake of monomeric substances have likely underestimated an important fraction of heterotrophic carbon cycling activity since the enzymatic systems used for selfish uptake are specifically tuned to their target high molecular weight substrates [6]. The prevalence of selfish uptake against a backdrop of changing bacterial community composition (Figs. 2, 4 and Figs. S2–S5), moreover, suggests that selfish uptake as a substrate acquisition strategy pays off sufficiently that it is comparatively widespread among bacteria. Selfish uptake is important not only in the surface waters of the ocean, but also in the upper mesopelagic and deep ocean, and is carried out by a wide range of heterotrophic bacteria whose carbon cycling activities help drive much of the marine carbon cycle.

## Supplementary information

Supplementary information

## Data Availability

Bacterial 16S rRNA gene sequences were archived as Illumina-generated libraries at the European Nucleotide Archive (ENA) of The European Bioinformatics Institute (EMBL-EBI) under the accession number PRJEB45894. Bacterial protein production data are available at DOI:10.26008/1912/bco-dmo.820556.1 FLA-PS hydrolysis data and cell counts are being submitted to BCO-DMO (https://bco-dmo.org/).
